# Evolving insights into the pleiotropic cardioprotective mechanisms of SGLT2 inhibitors

**DOI:** 10.1007/s00210-023-02459-9

**Published:** 2023-03-21

**Authors:** Anke C. Fender, Dobromir Dobrev

**Affiliations:** Institute of Pharmacology, West German Heart and Vascular Center, University Duisburg-Essen, Hufelandstrasse 55, 45147 Essen, Germany

The youngest type of antidiabetic agents, the so-called gliflozins or flozins, appear to have multifaceted pleiotropic effects. Canagliflozin was the first of the selective sodium-glucose cotransporter type 2 inhibitors (SGLT2i) to be approved, followed by empagliflozin, dapagliflozin, and others, to lower blood glucose in patients with type 2 diabetes (T2D). Unexpectedly, they also reduce cardiovascular mortality and the need for hospitalization in patients with heart failure (HF), regardless of the presence or absence of T2D. Accordingly, in 2022, the American College of Cardiology/American Heart Association Joint Committee on Clinical Practice Guidelines revised the treatment guidelines for the management of HF (Heidenreich et al. 2022) by including SGLT2i in their recommendations. Among the top ten take-home messages of the amended guidelines was the Class 2a recommendation for SGLT2i for therapy of HF with both preserved or mildly reduced ejection fraction (HFpEF, HFmrEF). Traditional approaches, including the beta-blockers and inhibitors of the renin–angiotensin–aldosterone system, obtained only a Class 2b recommendation.

The cardioprotection afforded by SGLT2i is notably independent of the glucose-lowering effect, and this, together with the largely absent expression of SGLT2 in most cell types present in the heart, presents a clear case of pleiotropic, off-target actions that are incompletely understood (Chen et al. 2022; Packer 2022). Insights into the mechanistic basis of SGLT2i actions were recently gained by applying deep learning AI to artificial neural networks (Bayes-Genis et al. 2021). The SGLT2i were predicted to reduce oxidative stress in cardiomyocytes and to decrease stiffness, extracellular matrix remodeling, concentric hypertrophy, and systemic inflammation most likely through inhibition of the Na^+^/H^+^ exchanger 1 (NHE-1). Intracellular Na^+^ accumulation is a key feature of failing cardiomyocytes, linked with derailed homeostasis and loss of contractility. Experimental and clinical evidence suggests that SGLT2i lower intracellular Na^+^ in cardiomyocytes, again independently of effects on glucose homeostasis (Baartscheer et al. 2017; Karg et al. 2018; Uthman et al. 2018). Molecular docking studies have confirmed the ability of the SGLT2i dapagliflozin to bind both NHE-1 and the Na^+^-Ca^2+^ exchanger (Yu et al. 2021). Elegant studies in cell-based and in vivo mouse models of ischemia and reperfusion (I/R) revealed that dapagliflozin could alleviate inflammatory stress by reducing Na^+^ and Ca^2+^ overload, protect lysosome integrity and function, and promote autophagosome degradation (Yu et al. 2021). Augmented autophagic flux appears to constitute the main mechanism by which SGLT2i improve cardiomyocyte homeostasis and metabolic flexibility (Madonna et al. 2023; Packer 2020, 2022; Wang et al. 2022, 2020; Yu et al. 2021). Finally, SGLT2i may also cause a shift in the transcriptional landscape of cardiomyocytes that mimics nutrient and oxygen deprivation, leading to activation of the autophagy-regulating platforms adenosine monophosphate-activated protein kinase (AMPK), the sirtuins SIRT1 and SIRT3, and hypoxia-inducible factors (HIF)-1α/2α, among others.

One important target for autophagosome phagocytosis and clearance in response to SGLT2i is the intracellular alarmin sensor NLRP3 (NLR family pyrin domain containing 3). The NLRP3-containing inflammasome is a multiprotein platform for the auto-activation of caspase-1, leading to the maturation of pro-interleukins (IL)-1β and IL-18. Caspase-1 also cleaves gasdermin D, a prerequisite for the formation of a pyroptotic pore through which IL1-β and IL-18 are secreted and propagate the inflammatory signal. Dapagliflozin applied prior to I/R in mice improved autophagic flux and reduced the activation of the NLRP3 system. Accordingly, cardiac caspase-1 activity and serum IL-1β levels were reduced, which was associated with a decrease in infarct size and serum marker enzymes (Yu et al. 2021). Numerous other studies corroborate the alleviation of NLRP3/caspase-1/IL-1β-dependent inflammatory stress by SGLT2i in various preclinical (Day et al. 2020; Dror et al. 2017; Philippaert et al. 2021; Sukhanov et al. 2021; Yan et al. 2022; Ye et al. 2017) and clinical settings (Kim et al. 2020).

In this issue of *Naunyn Schmiedebergs Archives of Pharmacology*, Hu and colleagues (Hu et al. 2023) report that the SGLT2i dapagliflozin ameliorates NLRP3 inflammasome activity in a mouse model of doxorubicin (DOX)-induced dilated cardiomyopathy (DCM). In this study, mice given 4 doses of 5 mg/kg DOX over 1 month displayed the typical anthracycline-induced ventricular dilatation and functional impairment, along with transcriptional priming of the NLRP3 inflammasome components *Nlrp3*, *Casp1*, and *Asc*, and augmented abundance of myocardial IL-1β, IL-18, and IL-6. Dapagliflozin co-treatment abrogated these features of inflammatory cardiomyopathy, an effect that could be verified in vitro. H9c2 myoblasts upon 24 h exposure to DOX transcriptionally primed NLRP3, pro-caspase-1, and pro-IL-1β, and accumulated and secreted mature IL-β, all of which were blunted by co-treatment with dapagliflozin. SGLT2i therefore appear to intervene in both priming and triggering (assembly) of the active inflammasome complex. This reflects similar findings made in the mouse atrial cardiomyocyte HL-1 cell line (Quagliariello et al. 2021), where empagliflozin counteracted DOX-driven NLRP3 expression.

To further elucidate the molecular mechanisms of dapagliflozin in H9c2 cells, the authors applied various selective inhibitors and could tentatively implicate regulation of toll-like receptor 4 (TLR), secondary to suppressed activity of the mitogenic kinases ERK and p38-MAPK. How the cross-talk between the signaling platforms occurs, or its temporal nature, was not clarified and should be addressed in future work. One interesting aspect of the present study by Hu et al. (Hu et al. 2023) is that AIM2 (*absent in melanoma 2*) protein, the central component of a different inflammasome platform, was also upregulated in myocardium of DOX-treated mice and normalized by co-administered dapagliflozin. AIM2 is a sensor for cytosolic DNA of pathogenic or mitochondrial origin (Bae et al. 2019). DNA binding resolves the inhibited state of AIM2, thereby triggering the assembly of the AIM2-containing inflammasome and resulting in the same caspase-1/cytokine maturation/pyroptosis cascade as the NLRP3 inflammasome (Sharma et al. 2019). Although the AIM2 inflammasome has been linked with myocardial infarction, viral myocarditis, diabetes-induced end-organ damage, and HF (Durga Devi et al. 2017; Onodi et al. 2021; Wang et al. 2019; Yu et al. 2020), to the best of our knowledge, Hu et al. provide the first evidence that this signaling platform is targeted by SGLT2i.

The novel observation that SGLT2i may inhibit signaling through NLRP3- and AIM2-containing inflammasomes has wider implications not only for cardiomyopathy and HF, but also for atrial fibrillation (AF). Recent meta-analyses and critical reviews of the large outcome trials (Fernandes et al. 2021; Li et al. 2021; Okunrintemi et al. 2021; Zheng et al. 2022) consistently reveal a lower rate of incident AF in the SGLT2i treatment arms. Empagliflozin was demonstrated to improve calcium handling in mouse atrial cardiomyocyte HL-1 cells via modulation of NLRP3 (Quagliariello et al. 2021). While the NLRP3 inflammasome is an established contributor to AF (Dobrev et al. 2023; Fender et al. 2020; Scott et al. 2021; Yao et al. 2018), the role of AIM2 in this context is just emerging. A recent study strongly implicates AIM2 as a mitochondrial ROS sensor that links a high protein diet with mitochondrial dysfunction and AF (https://doi.org/10.1016/j.hrthm.2022.03.451; 2022). Whether and how SGLT2i could affect this AF-promoting pathway warrants further study.

Both AF and HF present complex clinical syndromes that severely compromise patient survival once asymptomatic structural disease or cardiomyopathy advance to the point where overt symptoms manifest and quality of life is compromised. Recognition of risk factors and targeted therapeutic intervention during early disease development are therefore essential for the prevention of disease progression and the reduction of morbidity and mortality. Thus, the evolution of complex cardiomyopathies that drive HF and AF requires further elaboration in order to identify prognostic biomarkers and implement evidence-based therapeutic approaches in clinical guidelines.
